# Case Report: Rare Systemic and Aggressive ALK-Positive Histiocytosis With Recurrent Pancreatitis Treating by *Alectinib*

**DOI:** 10.3389/fmed.2022.840407

**Published:** 2022-05-19

**Authors:** Yanchu Li, Changle Shi, Yu Wu, Mingmin He, Xueming Xia, Jie Liu, Yu Jiang

**Affiliations:** ^1^Department of Head and Neck Oncology, West China Hospital of Sichuan University, Chengdu, China; ^2^Department of Pathology, West China Hospital of Sichuan University, Chengdu, China; ^3^Department of Hematology, West China Hospital of Sichuan University, Chengdu, China

**Keywords:** case report, ALK-positive histiocytosis, pancreatitis, ALK inhibitor, distinct entity

## Abstract

ALK-positive histiocytosis (APH) is a rare and recently described, solitary or generalized, histiocytic proliferative disorder with a characteristic gene translocation involving the fusion of the ALK gene at chromosome 2p23. To date, only 25 cases of APH have been reported. The patient presented with multiple nodules in the lung, liver, gallbladder, pancreas, kidney, and skin rashes, along with recurrent pancreatitis and cholecystitis. The histiocytes from the lesion were positive for CD68 and ALK and negative for S100 and CD1α. A reduced dose of the ALK inhibitor *alectinib* was administered rather than the standard dose of *alectinib* or chemotherapy because of recurrent pancreatitis, which has not been previously reported in APH cases. After 18 months of follow-up, the patient was maintained on *alectinib*, and a partial response (PR) was achieved.

## Introduction

ALK-positive histiocytosis (APH) is characterized by clonal proliferation of histiocytes and can present as either solitary or systemic. A series of ten cases with novel types of solitary (4/10) and systemic (6/10) histiocytosis was reported by Chan et al. (1, 2). A previous immunophenotypic study revealed that histiocytes were positive for ALK and histiocytic markers (CD68 and CD163), variably for S-100, but negative for CD1a, CD207, and BRAF-V600E (2, 3). Meanwhile, *KIF5B-ALK* and *COL1A2-ALK* gene fusions were identified via next-generation sequencing-based multiplex PCR (2), especially the primary *KIF5B-ALK* gene fusion, which was the most frequent (1, 4). However, no correlation has been observed between gene fusion type and disease localization or dissemination (2, 5).

Here, we report a case of aggressive systemic APH with severe recurrent pancreatitis. Furthermore, previously reported cases are reviewed.

## Case Presentation

### Clinical Presentation

**Figure 1:**
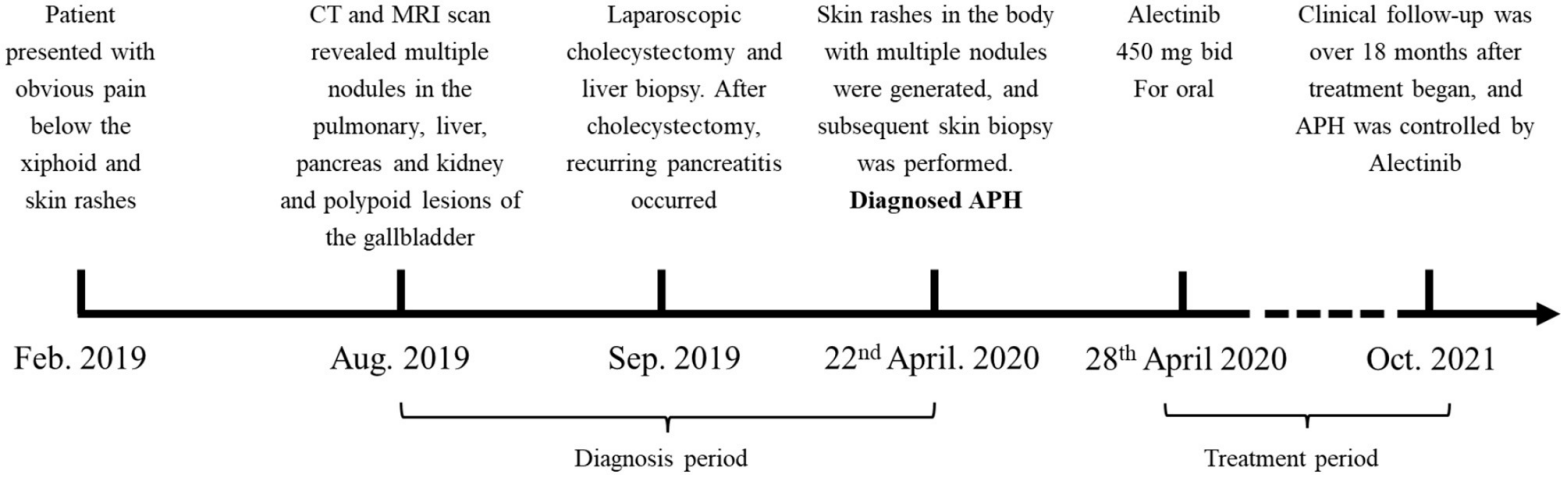
Treatment timeline. The patient suffered from APH since February 2019, and diagnosed in April 2020. After diagnosed, the patient was maintained on *alectinib* since the 28th April 2020. PR respond reached.

**Figure 2:**
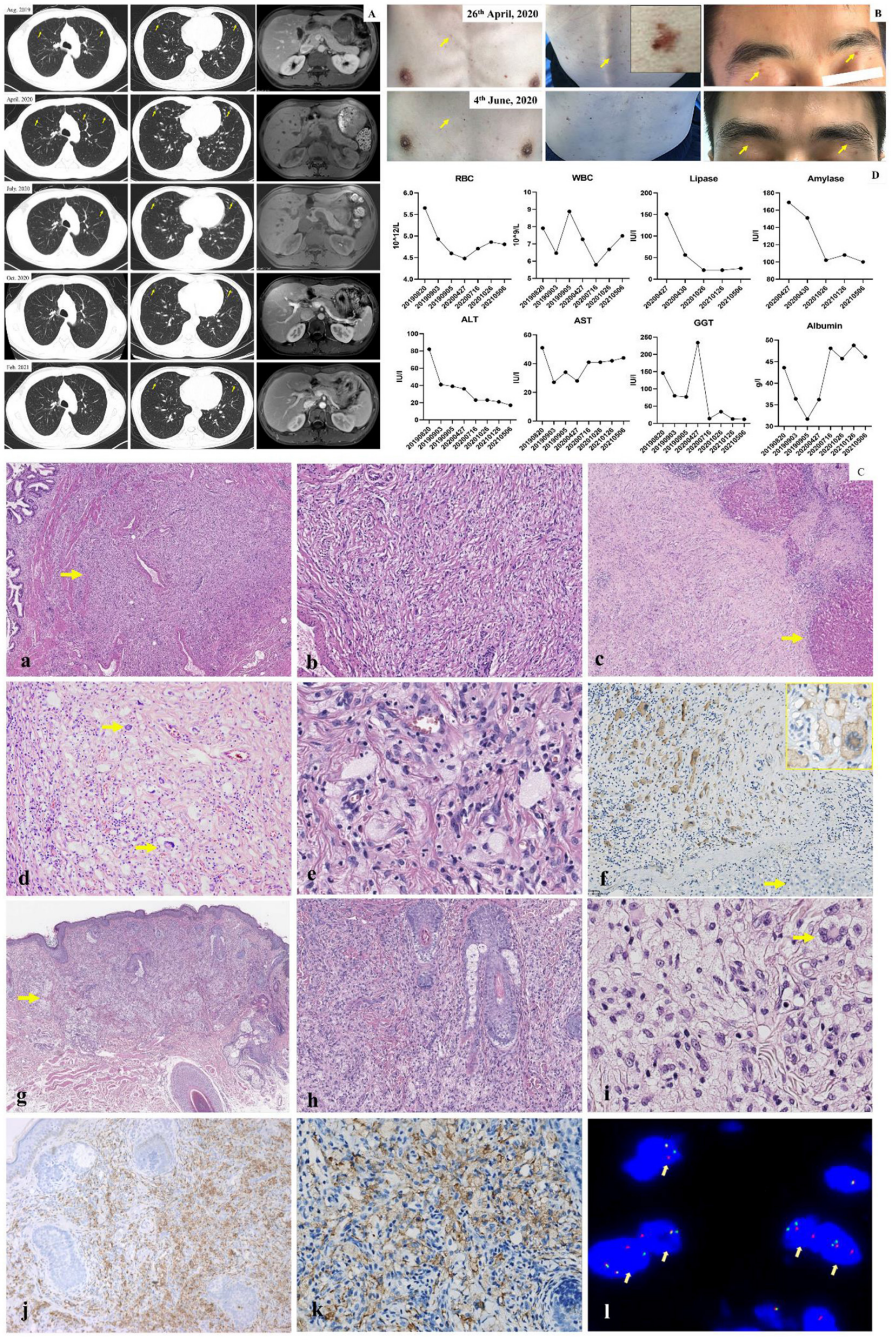
**(A)** CT and MRI scan of lung and abdomen. CT and MR images showed pulmonary nodule (yellow arrow), liver, kidney and polypoid lesion of gallbladder from August 2019 to February 2021. The multinodules resolved significantly following *alectinib* treatment. **(B)** Observation of skin lesion. Before treatment, the multiskin rash and nodule were observed (yellow arrow) on chest wall, back and face; After 1 month treatment, the multiskin rash and nodule were dramatically improved. **(C)** Pathology. (a) Nonencapsulated gallbladder lesion (arrow) with ill defined margin (H, E, 4x). (b) Gallbladder lesion of fascicular spindle cells, foamy histiocytes and inflammatory cells (H&E, 20x). (c) adjacent to liver capsule with infiltration of the liver parenchyma (arrow) (H&E, original magnification 4x). (d) Liver lesion of numerous foamy histiocytes admixed with Touton-type giant cells (arrow), spindle cells and inflammatory cells (H&E, 20x). (e) The histiocytes had small dark nuclei to medium-sized folded vesicular nuclei with fine chromatin and small to prominent nucleoli (H&E, 40x). (f) ALK expression with a cytoplasmic staining pattern in histiocyes and Touton-type giant cells (inset). Hepatocytes (arrow) were negative for ALK (20x, inset 40x). (g) Nonencapsulated skin lesion (arrow) with ill defined margin (H&E, 4x). (h) Xanthogranuloma-like appearance of the skin lesion (H&E, 10x). (i) Classic and characteristic moderate- to large-sized foamy histocytes with folded nuclei and small to prominent nucleoli of the liver lesion. Note also a Touton-type giant cells (arrow) (H&E, 40x). (j) CD68 expression in histiocytes of the skin lesion (20x). (k) ALK expression in histocytes of the skin lesion (40x). (l) Break-apart fluorescence *in situ* hybridization (FISH) assay for ALK gene rearrangements showed positive result with separations of the green and red signals (arrow, 100x). **(D)** Routine blood test, liver function and lipase/amylase test. No severe liver function, gastrointestinal and hematological toxicity over Grade II (CTCAE 3.0) were observed. For liver function, the levels of AST, ALT, GGT, and albumin were 28 U/l (0–40 U/l), 36 U/l (0–50 U/l), 234 IU/l (<60 IU/l), and 36.2 g/l (40–55 μmol/l), respectively. For routine blood test, WBC and RBC were 7.27 × 10^9^/l (3.6–9.5 × 10^9^/l) and 4.48 × 10^12^/l (4.3–5.8 × 10^12^/l), respectively. For lipase/amylase test, the lipase and amylase were 151 IU/l (13–60 IU/l) and 169 IU/l (344–135 IU/l), respectively. The recurrent pancreatitis was simultaneously cured and was accompanied by lipase/amylase normalization.

The treatment timeline is shown in Figure 1. A 32-year-old Chinese man presented with obvious pain below the xiphoid and skin rashes. CT and MRI scan revealed multiple nodules in the pulmonary system, liver, pancreas, and kidneys, and polypoid lesions of the gallbladder (Figure 2A). The patient underwent laparoscopic cholecystectomy and liver biopsy. After cholecystectomy, recurrent pancreatitis occurred, and skin rashes in the body with multiple nodules developed (Figure 2). Therefore, a subsequent skin biopsy was performed. At the beginning of treatment, the patient also had hypoproteinemia (albumin 31.7 g/l), pancreatitis (lipase 151 IU/l, amylase 169 IU/l), and liver dysfunction. Liver enzymes, including glutamic-pyruvic transaminase, aspartate aminotransferase (AST), and glutamine transpeptidase (GGT), increased to 82 IU/L (normal range, <50 IU/L), 51 IU/L (normal range, <40 IU/L), and 146 IU/L (normal range, <60 IU/L), respectively. However, hemoglobin, white blood cells (WBCs), red blood cells (RBCs), thrombocytes (PLT), and conjugated/unconjugated bilirubin levels were normal (Figure 2).

### Pathology

Microscopically, as shown in Figure 2C, lesions in both the gallbladder and liver were not encapsulated and had ill-defined margins. The lesion of the gallbladder and liver were shown in Figure 2Ca–c. Both lesions were composed of slender bland spindle cells with a fascicular growth pattern and were mixed with numerous moderate- to large-sized foamy histiocytes, a few Touton-type giant cells, lymphocytic infiltrates, eosinophils, and occasional plasma cells (Figure 2Cd). The histiocytes had small dark nuclei to medium-sized folded vesicular nuclei with fine chromatin and small to prominent nucleoli (Figure 2Ce). No mitosis or necrosis was observed in either of the lesions. Immunohistochemically, the lesion cells showed cytoplasmic staining for ALK (Anti ALK clone OTI1H, 1:100, Zhongshan Golden Bridge Biotechnology, Beijing, China) (Figure 2Cf), and the histiocytes were positive for CD68 (CD68-PGM-1, Prediluted, Zhongshan Golden Bridge Biotechnology, Beijing, China) but negative for CD1α (CD1α-EP80, 1:200, Zhongshan Golden Bridge Biotechnology, Beijing, China), S-100 protein (S100-4C4.9, 1:300, MXB Biotechnology, Fuzhou, China), and Langerin (Langerin-12D6, Prediluted, MXB Biotechnology, Fuzhou, China). Spindle cells were positive for SMA (SMA-UMAB237, 1:200; Zhongshan Golden Bridge Biotechnology, Beijing, China). BRAF V600E and V600K mutations, tested via PCR and Sanger sequencing, were negative. Break-apart fluorescence *in situ* hybridization (FISH) assay for *ALK* gene rearrangements revealed split signals in 30% of the analyzed nuclei. Thus, the initial diagnosis of an inflammatory myofibroblastic tumor was made, which seemed to be inconsistent with the involvement of multiple organs in the patient. The skin biopsy taken subsequently revealed a xanthogranuloma-like lesion (Figure 2Cg,h), composed of large foamy histiocytes and Touton-type giant cells (Figure 2Ci), similar to those seen in the liver and gallbladder lesions. The histiocytes were positive for CD68 (Figure 2Cj), CD163, and ALK (Figure 2Ck) but negative for the S-100 protein. FISH assays were positive for *ALK* rearrangements (Figure 2Cl). This, a final diagnosis of APH was made.

### Treatment

At the beginning, the patient was not provided specific treatment, which not only resulted in the rapid and continuous increase of both lesion size and the number of nodules in the involved organs (Figure 2) but also caused recurrent pancreatitis and body weight loss of approximately 15 kg. However, because of recurrent pancreatitis, chemotherapeutic agents and ALK inhibitors that increase the risk of pancreatitis, such as *ceritinib* and *crizotinib*, could not be used for treatment. Thus, the ALK inhibitor *alectinib* (450 mg bid) was used, then this patient was maintained on *alectinib*. After treatment, the multiple nodules in the pulmonary system, liver, pancreas, kidneys, as well as skin rashes were dramatically improved (Figure 2), and partial response was achieved. Clinical follow-up was conducted after treatment initiation, and revealed that APH progression was inhibited by *alectinib*. The recurrent pancreatitis was simultaneously cured and was accompanied by lipase/amylase normalization (Figure 2D). Meanwhile, according to the Common Terminology Criteria for Adverse Events (CTCAE) criteria, no severe liver function, gastrointestinal. or hematological side effects were observed beyond grade II.

The patient was still followed-up and remained on *alectinib* treatment. There was no evidence of APH recurrence, and the pancreatitis was cured and had no recurrence.

## Discussion

APH is a rare disease; the first publication on APH was in 2008 (1). Currently, classification systems of histiocytosis have evolved into five groups, which are defined based on the cell of origin, molecular mutations, and clinical behavior (5). However, APH has not yet been included in the WHO classification of tumors (6). Recently, only 26 cases, including this study, have been reported under the term “APH” (1–4, 7–17).

The pathogenesis of this condition is yet to be fully elucidated. The general information, histological and clinical characters of the 26 patients is shown in Tables 1, 2. The male-to-female ratio was 9:17, and systemic and solitary cases were equal. The median age of the patients was 15 years. Regarding race, 6 patients were *Caucasian*, 8 were *Asian*, including 4 Chinese patients (1, 2, 8), and the other 12 were not stated. Histologically, the tumor histiocytes were large, with irregularly folded, lobulated, or clefted nuclei, fine chromatin, and abundant eosinophilic or foamy cytoplasm, sometimes with emperipolesis (3, 10). In some cases, the tumor cells displayed a spindle appearance with a fascicular to storiform growth pattern (3). Touton-type giant cells and lymphocytic infiltrates were observed. Immunohistochemically, the histiocytes were positive for ALK and histiocytic markers (CD68 and CD163), variable for S-100 and negative for CD1a, CD207, and BRAF-V600E. The staining pattern for ALK can be cytoplasmic, membranous, and perinuclear or cytoplasmic dot-like (14). *ALK* rearrangement was identified in all APH cases, and *KIF5B-ALK* was the most frequent gene translocation, and the detailed information on the gene types of the 26 patients is shown in Table 2.

**Table 1:** Summary of clinicopathologic findings for patients with solitary and systemic APH.

**Solitary APH: 13 cases**						
**Gender**		**Age (Year)**		**Organ involved**		
**Male**	**Female**	**>** **3**	**>18**	
				Breast		3 cases
4	9	92.4% (12/13)	53.8% (7/13)	Skin or subcutaneous soft tissue	Nasal skin	1 case
					Foot	1 case
					Umbilicus	1 case
				Intracranial	Cavernous sinus	1 case
					Cerebellar vermis	1 case
					Right pericentral region	1 case
				Intraabdominal	Appendix	1 case
					Mesentery	1 case
				Lung		1 case
				Intradural extramedullary		1 case
**Systemic APH: 13 cases**						
**Gender**		**Age (Year)**		**Organ involved**	
**Male**	**Female**	**<3**	**>16**	
5	8	69.2% (9/13)	30.8% (4/13)	Bone marrow, Liver, spleen (7/13)	+ None	4 cases
					+ Peripheral blood	1 case
					+ Skin	1 case
					+ Kidney	1 case
				Bone marrow, Liver	+ Kidney, lung, skin	1 case
				Bone marrow	+ Intestine, CNS	1 case
				Breast	+ Bone	1 case
				Pancreas, lung (3/13)	+ Breast, brain	1 case
					+ Liver, skin, gallbladder, kidney	1 case
					+ Liver, prostate, parotid gland, bone,	1 case
					brain, peripheral blood

**Table 2:** Summary of organ involvement, gene type, treatment and response for patients with APH.

**Case No**.	Ref^*^	**Sex**	**Age**	**Organs involved**	**ALK abnormality**	**Treatment**	**Follow-up**	Res.^*^
1	1	F^*^	Neonate	Liver, spleen, skin, bone marrow	TPM3	Dexamethasone & etoposide	12 y^*^	PR^*^
2	1	F	3 mo^*^	Liver, spleen, bone marrow	ALK-positive by IHC	Dexamethasone & etoposide	14 y	CR^*^
3	1	F	3 mo	Liver, spleen, bone marrow	ALK-positive by IHC	Antibiotics	13 y	CR
4	10	F	0 mo 27 d^*^	Liver, spleen, bone marrow, kidney	ALK rearrangment	Prednisone& vinblastine	7 y	PD^*^
5	10	M^*^	0 mo 2 d	Liver, spleen, bone marrow	ALK-positive by IHC	Cytarabine-based Chemotherapy	Unclear	SD^*^
6	2	F	2 m	Liver, spleen, bone marrow	KIF5B	Vinblastine & etoposide	2 y	CR
7	2	M	3 mo	Liver, skin, lung, bone marrow, kidney	KIF5B	Chemotherapy (ALCL99 protocol)	4 y	PR
8	2	M	2 y 9 mo	Intestine, bone marrow, CNS	KIF5B	Etoposide, cyclosporine, MTX	2 m	Died
9	2	M	2 y 3 mo	Nasal skin	KIF5B	Incomplete excision	18 m	CR
10	2	M	15 y	Cavernous sinus	KIF5B	Crizotinib	6 M	CR
11	2	M	16 y	Skin & soft tissue of foot	KIF5B	Surgical resection	3 y	CR
12	2	F	40 y	Breast	COL1A2	Surgical resection	3.5 y	CR
13	9	F	50 y	Appendix	KIF5B	Surgical resection	Limited follow-up	CR
14	15	F	Neonate	Liver, spleen, bone marrow, peripheral blood	ALK-positive by IHC	Sulfamethoxazole/ Trimethoprim	6m	PR
15	13	F	7 y	Cerebellar vermis	KIF5B	Surgical resection	1 y	CR
16	13	F	10 y	Pericentral cortical	KIF5B	Surgical resection	6 mo	CR
17	11	M	27 y	intradural extramedullary, nerve root (L3)	KIF5B	Surgical resection	9 mo	CR
18	16	F	37 y	Breast & bone (ECD-like)	ALK-positive by IHC	Ibrutinib	4 y	CR
19	14	M	49 y	Lymph node, lung, liver, pancreas, prostate, bone, cutanous brain lesion, peripheral blood	KIF5B	γ-knife, anti-VEGF/PD-1/ALK	3 mo- 2 mo	PD-PR
20	4	F	20 y	Mesentery	TRIM33	Surgical resection	1 y	CR
21	8	F	52 y	Lung	EML4	Surgical resection	5 mo	CR
22	11	F	45 y	Breast	KIF5B	Surgical resection	1 mo	/
23	12	F	38- y	Breast	KIF5B	Surgical resection	1 mo	/
24	12	F	16 y	Breast, brain lesion, pancreas, lung	KIF5B	Surgical resection & Alectinib	33 mo	CR
25	3	F	18 y	Umbilicus	KIF5B	Surgical resection	18 mo	CR
26	Current	M	32 y	Lung, liver, pancreas, skin, gallbladder, kidney	ALK rearrangment	Alectinib	27 mo	PR

*F, Female; M, male; y, year; mo, month; d, day; CR, complete response; PR, partial response; SD, stable disease; PD, progressive disease; Res, result; Ref, references.

Meanwhile, APH should be differentiated from other histiocytosis types, including juvenile xanthogranuloma (JXG), Erdheim-Chester disease (ECD), Langerhans cell histiocytosis, and Rosai-Dorfman disease (RDD) (18–20). The tumor histiocytes with marked nuclear foldings, which are not found in other histiocytosis types, could be a clue for APH (10), and an immunohistochemical profile featuring positivity to both CD68 (CD163) and ALK virtually excludes all the histiocytic neoplasms as well as all the other ALK-positive neoplasms, such as ALK-positive peripheral T cell lymphoma, ALK-positive B cell lymphoma, lung adenocarcinoma, inflammatory myofibroblastic tumor, and some melanocytic tumors of the Spitz lineage (1). Notably, some cases of histiocytosis with *ALK* gene rearrangement are reported under the diagnosis of ECD, JXG, atypical juvenile histiocytosis, or histiocytosis not otherwise specified (11, 12, 21), some of which have detailed clinicopathologic data displaying the lack of characteristic long bone or skin involvement for ECD or JXG, respectively (12). In addition, Huang et al. reports two cases of congenital/ early-onset RDD without any molecular detection for *ALK*, yet displaying an identical clinical presentation and proliferation in young children (10). These reported cases reveal that whether APH is a distinct entity is unclear and controversial. However, as illustrated by Chen (1, 2) and other researchers (3, 4, 7–16), APH differs significantly from ECD, JXG, and RDD in terms of clinical, morphological, and genetic aspects.

The clinical features of the previously reported cases of APH are summarized in Table 2. Surgery is the typical treatment for localized APH. A total of 92.3% (12/13) of the localized cases underwent surgical resection and were followed up from 1 month to 3.5 years after surgery, and no recurrence was found. One patient showing involvement of the cavernous sinus was treated with the ALK inhibitor *crizotinib*, and a *complete response* (CR) was reached 3 months later. No recurrence was observed for 6 months after follow-up. On the other hand, treatment for systemic APH mainly includes chemotherapy and/or ALK inhibitors (8, 16). Thus, 8 patients received chemotherapy, of which 37.5% (3/8) received chemotherapy alone, 50.0% (4/8) received steroid therapy, and 12.5% (1/8) was treated with the PD-1 inhibitor *pembrolizumab*. Moreover, previous clinical studies have shown an encouraging response of the cavernous sinus, pancreas, and lung lesions to ALK inhibitor treatment (2, 8); thus, even if chemotherapy is not effective, PR and CR might be achieved by receiving anti-ALK therapy as second-line therapy. More recently, *ibrutinib*, a Bruton tyrosine kinase (BTK) inhibitor, was shown to be effective in and was able to achieve CR from rare APH cases with concurrent CLL/SLL.

For this systemic APH patient with severe recurrent pancreatitis, not only was the patient unable to tolerate the standard treatment; the patient's disease also had an aggressive biological behavior and progressed rapidly. The ALK inhibitor *alectinib*, which did not induce pancreatitis, was selected, and a 25% reduced dose was used (12, 14).

To our knowledge, this is the first report of a patient with systemic and aggressive APH with severe recurrent pancreatitis. Because of recurrent pancreatitis, the treatment strategy is different from common APH cases since the disease is more aggressive. Thus, in summary, we indicated that APH is not just an indolent disease and that those complex complications represent APH as an aggressive disease. Moreover, the original report was published 13 years ago, but APH entities were likely under-reported.

## Data Availability Statement

The raw data supporting the conclusions of this article will be made available by the authors, without undue reservation.

## Ethics Statement

Ethical review and approval was not required for the study on human participants in accordance with the local legislation and institutional requirements. Written informed consent for participation was not required for this study in accordance with the national legislation and the institutional requirements. Written informed consent was obtained from the individual(s) for the publication of any potentially identifiable images or data included in this article.

## Author Contributions

YJ and YL contributed to the study conception and design. YL, CS, YW, and MH performed material preparation, data collection, and analysis. YL, XX, and JL wrote the first draft of the manuscript. All authors commented on previous versions of the manuscript and read and approved the final manuscript.

## Conflict of Interest

The authors declare that the research was conducted in the absence of any commercial or financial relationships that could be construed as a potential conflict of interest.

## Publisher's Note

All claims expressed in this article are solely those of the authors and do not necessarily represent those of their affiliated organizations, or those of the publisher, the editors and the reviewers. Any product that may be evaluated in this article, or claim that may be made by its manufacturer, is not guaranteed or endorsed by the publisher.
